# Relationship between occupational and leisure-time physical activity and the need for recovery after work

**DOI:** 10.1186/s13690-022-01017-8

**Published:** 2023-02-10

**Authors:** Tiina Karihtala, Anu M. Valtonen, Hannu Kautiainen, Leila Hopsu, Janne Halonen, Ari Heinonen, Sampsa Puttonen

**Affiliations:** 1 Faculty of Sport and Health Sciences, University of Jyväskylä, Jyväskylä, Finland; 2 Metropolia University of Applied Sciences, Helsinki, Finland; 3 Primary Health Care Unit, Kuopio University Hospital, Kuopio, Finland; 4 Folkhälsan Research Center, Helsinki, Finland; 5 Myontec Oy, Kuopio, Finland; 6 Finnish Institute of Occupational Health, Helsinki, Finland; 7 Faculty of Sport and Health Sciences, University of Jyväskylä, Jyväskylä, Finland; 8 Faculty of Social Sciences, University of Tampere, Tampere, Finland; 9 Finnish Institute of Occupational Health, Helsinki, Finland

**Keywords:** Occupational physical activity, Leisure-time physical activity, Need for recovery after work, Accelerometer

## Abstract

**Background:**

Health benefits of physical activity are very well acknowledged but the role of both occupational physical activity (OPA) and leisure time physical activity (LTPA) in recovery after work is not thoroughly understood. The purpose of this study was to investigate the association between accelerometer-measured OPA and LTPA and the need for recovery after work (NFR) in early childhood education and care (ECEC) professionals.

**Methods:**

The study participants were 217 female ECEC professionals aged 17–64. Physical activity was recorded with a three-axis accelerometer (ActiGraph GT9X Link, ActiGraph, USA) for seven consecutive days. Separate analyses were conducted for both OPA and LTPA and reported as hours/day based on different intensity levels (light, moderate, vigorous, very vigorous). The NFR was measured with the Need For Recovery (NFR) scale (0%–100%).

**Results:**

Participants’ average physical activity for both OPA and LTPA was about 4 h/day, and the mean NFR score was 38.4%. OPA was significantly associated with the NFR but not with LTPA. The relationship remained significant after adjustments for age, body mass index, work ability, mental health status, and sleep difficulties (*p* < 0.024).

**Conclusion:**

According to this study, the OPA level is related to the level of the NFR in female ECEC professionals. Based on the results, it seems that LTPA has no relevance to the NFR. Results suggest that long-lasting OPA, even without strenuous physical activity at work, may predispose individuals to a high NFR.

## Background

The vast health benefits of physical activity (PA) are very well acknowledged [10, 37, 38, 41, 46]. However, not all PA seems to be beneficial to health and wellbeing [8, 26]. High leisure-time physical activity (LTPA) has been reported to improve work ability [21, 29] and protect from early retirement [36], whereas high occupational physical activity (OPA) and a lack of LTPA are related to decreased work ability and early retirement [4, 6]. Decreased work ability is often preceded by a subjective feeling of overload. This phenomenon is introduced as the need for recovery after work (NFR) and refers to a feeling of overload and lack of energy after work. The NFR is described as an early indicator of fatigue at work. [55]. Recent studies suggest that a high NFR increases the likelihood of decreased work ability and early retirement [51, 52]. NFR assessment can be used as a preventive tool to track employee wellbeing [14, 49]. Therefore, it is important to identify the factors that can contribute to the NFR and thereby prevent decreased work ability, sickness absenteeism, and ultimately, early retirement.

The significance of PA in work ability (WA) is reported with inconsistent results [6, 54, 56]. This incoherence may be due to different methods to measure PA [54] or the absence of separation of OPA and LTPA. High OPA has been associated with low WA [56], but for example among nurses contradictory results have been reported [54]. Also, lack of LTPA is shown to associate with poor work ability [5, 21, 29, 42, 44]. Further, literature about the associations of both LTPA and OPA with the NFR is scarce. The relatively small body of literature on the association between LTPA and the NFR suggest that high LTPA may result in a lower NFR [11, 57], and LTPA may help individuals to detach from their work and hence enhance their recovery [28]. Also, physical activity, especially outdoors, is reported to decrease NFR [35]. A high level of OPA is reported to be related to a high NFR in cleaning, manufacturing, and transportation sectors [51] as well as among office workers, occupational health physicians and managers [34]. However, the results have been inconsistent across sectors and Gommans et al. [20] reported that high OPA was related to NFR in industry workers, but not in healthcare workers. Also, among white-collar workers, a higher OPA level was associated with a lower NFR [11]. A systematic understanding of how one’s OPA level contributes to the NFR is still missing. Furthermore, to our knowledge no research on the relation of NFR and OPA and LTPA among early childhood education and care (ECEC) center professionals exist.

Currently, knowledge about the associations between LTPA or OPA and the NFR is predominantly resting on self-reports and questionnaire-based instruments to measure PA [11, 16, 39] and only few have used objective measures of PA [51]. PA levels measured with self-report methods have been shown to differ significantly from objective measures [50]. Inconsistent results between NFR and OPA and LTPA may thus at least partly result from different methodology that fail to measure for example diverse working conditions [54]. It has been recommended that technical instruments should be used to ensure objectivity in PA measurement [15].

There is a limited number of research on the significance of both OPA and LTPA on NFR. Existing results are lacking congruence and knowledge is mainly based on self-reported OPA and LTPA. Further, knowledge of how PA and work-related factors are linked among early childhood education and care (ECEC) center professionals is almost non-existent. More specifically, no information exists on how OPA and LTPA are associated with the NFR among ECEC professionals. Therefore, the aim of this study is to investigate the association between accelerometer-measured OPA and LTPA and the NFR in ECEC professionals.

## Methods

### Design and participants

This cross-sectional study is part of the DagisWork study (workplace healthcare interventions to promote the work ability of kindergarten personnel). The study was conducted in ECEC centers in two cities in Southern Finland during the period 2017–2019. From a total of 218 centers in the area, 78 volunteered to participate in the study, and a random sample of 23 centers was chosen. In total, 386 participants were recruited from these ECEC centers. Pregnancy, temporary employment, or retirement during the following six months were used as exclusion criteria. Thus, 269 participants (aged from 17 to 64, 99% women and 1% men) signed informed consent forms and joined the study. The participants completed an online questionnaire. Two experienced healthcare professionals measured height and weight and calculated body mass index (BMI, kg/cm^2^) and instructed the baseline measurements at each ECEC center premises during working hours. Over the following seven days, the participants’ PA was recorded. Only participants with full NFR and PA data were included in the analysis. Men were excluded because of their limited number (*n* = 3). Finally, 217 participants were included in the analysis.

### Questionnaire

During the onsite measurements, participants received a personal link to an online questionnaire, which included questions on their background characteristics. Participants were classified to smokers or non-smokers and alcohol consumption was measured as units/week (1 unit = 12 g of pure alcohol). The self-rated health was measured with a five-point Likert-scale (good, somewhat good, average, somewhat poor, poor). Education level was also inquired: no vocational training, vocational training, bachelor’s level or master’s level. Marital status was classified as living in partnership or not, and years working in ECEC centers was inquired.

*Work ability* was measured with a single-item question on one’s current perceived work ability compared with one’s lifetime best using a numeric scale of 0–10 (0 = completely unable to work; 10 = work ability at its best) [32].

The *Perceived Stress Scale (PSS)* was used to measure an individual’s stress level [12]. This 14-item instrument evaluates how often different situations in one’s life are experienced as being stressful, for example, “In the past month, how often have you felt nervous or stressed?” Each item was rated on a 5-point scale (0 = never; 1 = almost never; 2 = sometimes; 3 = fairly often; 4 = very often). Positive items were reversed, and a sum score was calculated (0–56).

The *General Health Questionnaire (GHQ-12)* was used to assess mental health status [19]. Responses were given on a 4-point scale, and a Likert-scoring method was used to calculate the sum scores (0–36). The higher the score, the more severe the mental health condition.

*Sleep difficulties* during the last four weeks were measured using the Jenkins Sleep Scale (JSS) [33] and included difficulties in falling asleep, waking up several times each night, difficulties in staying asleep, and feeling tired and worn out after waking up after one’s usual amount of sleep. The response choices ranged from 1 = “not at all” to 6 = “every day,” and the points were added up to give a total (4–24), with a higher score indicating more sleep difficulties.

*Disorders* were assessed with an open question, and the responses were dichotomized (yes/no). Only disorders diagnosed by a doctor and reported to be present or emerging often or repeatedly were classified as “yes” in the data (musculoskeletal, cardiovascular, respiratory, and mental disorders).

*Need for recovery after work.* The NFR was measured with the Need For Recovery scale, which has been reported to be a valid tool for measuring the acute need for recovery and indicating possible future fatigue [55]. The scale consists of 11 dichotomous claims, such as ‘‘I find it hard to relax at the end of a working day,’’ “I find it difficult to concentrate in my free time after work,” and “By the end of the working day, I feel really worn out.” Participants gave “yes” or “no” answers, and scores were calculated as a percentage of the positive answers for the participants who answered at least 8 out of the 11 questions. The final scores varied between 0 and 100, and the higher the score, the higher the NFR of the participant.

### Physical activity

Physical activity was recorded with a three-axis accelerometer (ActiGraph GT9X Link, ActiGraph, USA). In addition, participants filled in a diary to document their working hours and sleep times every day during the measurement period. Based on the diary data, each day’s awake time was divided into two different sets of PA as follows: PA during working hours (occupational physical activity = OPA) and PA during free time (awake time—working hours, i.e., leisure time = LTPA).

Participants used waist-worn PA monitors for seven consecutive days except when taking baths and showers or participating in other activities involving water. The analysis was conducted with ActiLife software (6.13.3) and 60-s epoch, and a 30 Hz frequency was used. The PA thresholds were set using the [18] cut points: sedentary: 0–99 counts per minute (cpm); light PA: 100–1951 cpm; moderate PA: 1952–5724 cpm; vigorous PA: 5725–9498 cpm; and very vigorous PA: over 9499 cpm. For moderate to vigorous PA (MVPA), the total sum of moderate, vigorous, and very vigorous PA was calculated, and at least ten consecutive minutes of activity was required for it to be recognized as MVPA. The non-wear time criteria from Choi et al. [9] were used, and for sedentary time, a minimum length of 30 min and a drop time of 2 min were established. For each activity level, the average minutes/day were calculated. All the participants with at least four days and ten hours/day of valid accelerometer data were included in the analysis. For the final analysis, all the intensity levels (light, moderate, vigorous, very vigorous) were added up for OPA and for LTPA.

### Statistical analysis

The descriptive statistics were presented as means with standard deviation (SD), as medians with interquartile range (IQR) or as counts with percentages. The linearity relationships across the three-level groups (tertiles) of Need For Recovery (NFR) were evaluated using the Cochran-Armitage test (chi-square test for trend), logistic models (dichotomous variables), Cuzick test (ordinal variables) and analysis of variance (ANOVA) with an appropriate contrast (orthogonal). Multivariate linear regression analysis was used to identify the relationship between Need For Recovery (NFR) and physical activity (PA) levels according to the occupational physical activity (OPA) and leisure time physical activity (LTPA) levels with standardized regression coefficient Beta (β). The Beta value is a measure of how strongly the predictor (FMI or LMI) variable influences the criterion variable. The Beta is measured in units of SD. Beta values were adjusted for age, BMI, and work ability (WA). Cohen’s standard for Beta values above 0.10, 0.30 and 0.50 represents small, moderate and large relationships, respectively [13]. The possible non-linear relationship between NFR (%) and OPA and LTPA values were modeled using restricted cubic splines regression models with 3 knots at the 25th, 50th, and 75th percentiles,knot locations are based on Harrell’s recommended percentiles [24]. Normal distributions were evaluated graphically and with the Shapiro–Wilk W test. Stata 17.0 (StataCorp LP, College Station, TX, USA) was used for the analysis.

## Results

### Characteristics of the study population

**Fig. 1:**
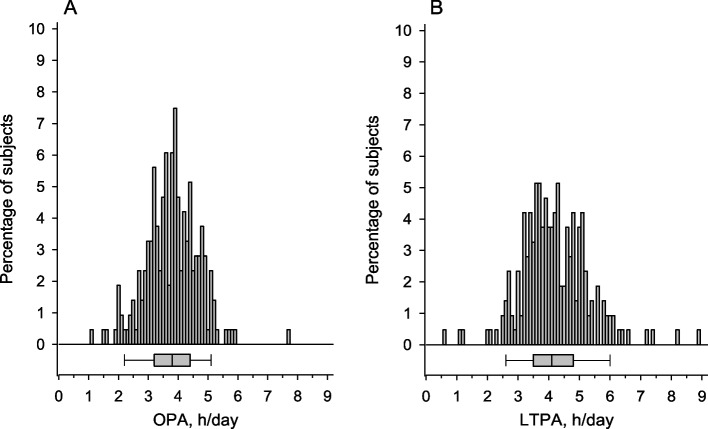
Distribution of the occupational physical activity (OPA) (panel **A**) and leisure time physical activity (LTPA) (panel **B**). Box-and-whiskers plot shows median with interquartile range (IQR), and whiskers indicate 5th and 95th percentiles of distributions

The participants (*n* = 217) represented 5 different ECEC professions (assistants: *n* = 9; child carers: *n* = 95; teachers: *n* = 83; special education teachers: *n* = 14; managers: *n* = 16) relevant to the typical distribution of personnel in ECEC centers in Finland. Participants average hours/day for OPA was 3.8 h (SD = 0.9) and for LTPA 4.2 h (SD = 1,1) (Fig. 1).

The mean NFR score for the study population was 38.4% (SD 26.2). The characteristics of the participants according to the NFR tertiles are presented in Table 1. Across the NFR tertiles, a statistically significant linear relationship was observed with work ability, self-rated health, the PSS, the GHQ-12, and the JSS.

**Table 1:** Demographic and clinical characteristics of the participants divided into tertiles according to the Need For Recovery (NFR)

	**NFR tertiles**			
	**I** **0–18%** ***N* = 71**	**II** **27–45%** ***N* = 74**	**III** ** ≥ 54%** ***N* = 72**	***P*-value**^**a**^
**Age, mean (SD)**	43 (11)	45 (12)	45 (10)	0.43
**In partnership, n (%)**	51 (72)	56 (76)	51 (71)	0.89
**Education, n (%)**				0.58
no vocational training	3 (4)	3 (4)	1 (1)	
vocational level	34 (49)	43 (58)	33 (46)	
bachelor’s level	15 (21)	15 (20)	21 (29)	
master’s level	18 (26)	13 (18)	17 (24)	
**Years spent working in an ECEC center, mean (SD)**	3.4 (0.8)	3.5 (0.8)	3.6 (0.7)	0.32
**Smoker, n (%)**	11 (15)	13 (18)	10 (14)	0.79
**Alcohol consumption/week**^**b**^**, median** (IQR)	1 (0,3)	1 (0,2)	1 (0,3)	0.69
**BMI, mean (SD)**	26.6 (6.7)	27.9 (6.6)	27.3 (5.7)	0.48
**Self-rated health, n (%)**				< 0.001
good	27 (38)	10 (14)	9 (13)	
somewhat good	27 (38)	30 (41)	32 (44)	
average	13 (18)	24 (32)	21 (29)	
somewhat poor	4 (6)	7 (9)	9 (13)	
poor	0 (0)	3 (4)	1 (1)	
**WA, mean (SD)**	8.7 (0.8)	8.1 (1.3)	7.7 (1.3)	< 0.001
**PSS, mean (SD)**	13.1 (5.2)	14.9 (4.8)	20.8 (6.3)	< 0.001
**GHQ-12, mean (SD)**	9.6 (3.4)	10.8 (3.8)	15.6 (5.3)	< 0.001
**JSS, mean (SD)**	9.8 (3.7)	10.7 (4.0)	12.9 (4.1)	< 0.001
**Disorders, n (%)**				
musculoskeletal disorders	13 (18)	26 (35)	16 (22)	0.60
cardiovascular disorders	9 (13)	11 (15)	14 (19)	0.27
respiratory disorders	7 (10)	8 (11)	12 (17)	0.22
mental disorders	3 (4)	8 (11)	5 (7)	0.54

*ECEC* Early childhood education and care, *IQR* Interquartile range, *BMI* Body mass index, *WA* Work ability, *PSS* Perceived Stress Scale, *GHQ-12* General Health Questionnaire, *JSS* Jenkins Sleep Scale

^a^p for linearity across Need For Recovery (NFR) tertiles

^b^Units per week (1 unit = 12 g of pure alcohol)

### Relationship between PA and the NFR

**Fig. 2:**
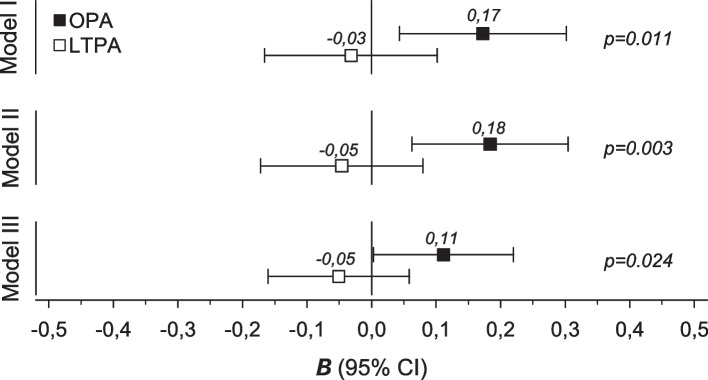
Relationship between the Need For Recovery (NFR) and occupational physical activity (OPA) and leisure time physical activity (LTPA) levels. Model I) crude, Model II) adjusted for age, BMI, and work ability (WA), and Model III) adjusted for age, BMI, work ability, the General Health Questionnaire (GHQ-12), and the Jenkins Sleep Scale. Values show beta coefficients. Cohen’s standards for beta (β) values above 0.10, 0.30, and 0.50 represent small, moderate, and large relationships, respectively

In Fig. 2 the association between the NFR and OPA and LTPA is illustrated with beta-coefficients and confidence levels. OPA, but not LTPA, had a significant but weak association with the NFR. The relationship with OPA was weak but remained significant after adjustments for age, BMI, WA, the GHQ-12, and the JSS (β = 0.11, 95% CI: 0.01 to 0.22) (Model III). No correlation was found between the levels of OPA and LTPA (*r* = 0.11, 95% CI: -0.03 to 0.24).

**Fig. 3:**
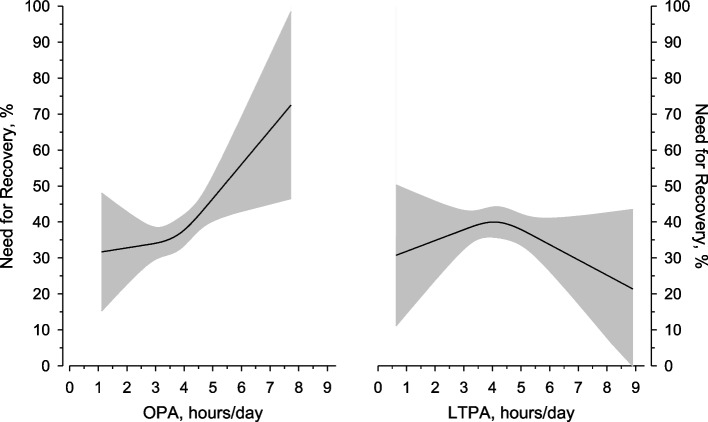
Relationship between the occupational physical activity (OPA) level and leisure time physical activity (LTPA) level (hours/day) and the Need For Recovery (NFR) (%). The curves were derived from a 3-knot restricted cubic splines regression models. The models were adjusted for age, BMI, and work ability. The 95% confidence intervals are represented as gray areas

Figure 3 illustrates the cubic spline regression of the NFR level in relation to the hours/day of OPA and LTPA. Positive relationship was found between OPA and NFR regression line showing that the higher OPA was, the more the participants experienced the NFR. All the same, this relationship was not seen between LTPA and the NFR.

## Discussion

The results of this study among female ECEC professionals suggest that the accelerometer-measured OPA level (average hours/day) is related to the level of the NFR. That is to say, the more physically active the employees were during their working hours, the higher their experienced NFR was. This relationship was independent of age, WA, BMI, mental health status, and sleep difficulties. Based on our results, LTPA appears not to have relevance to employees’ NFR.

Our observation of an association between OPA and the NFR is in line with the findings of Stevens et al. [51]. They also utilized objectively measured PA and reported an association between OPA and the NFR, especially with regard to higher intensity PA. Their relatively large sample comprised blue-collar workers from several fields, including cleaning, transportation, and manufacturing. However, Coffeng et al. [11] reported contradictory results in office workers, suggesting that reductions in the NFR could be achieved by performing more PA during working hours. These conflicting results may, at least partially, arise from different work content and the distinct nature of the work demands between the worker groups. For example, when predominantly sedentary work is interrupted with short breaks to perform PA, the result might be a lower NFR. Differences between sectors have also been reported, and for example, the relationship between OPA and the NFR seems to be stronger in industry sector workers than in healthcare sector workers [20]. Our results are in line with Gommans et al. [20] and it possible that the intensity and the level of OPA is quite similar in our population with ECEC professionals and health care workers. Both occupations include high level of light intensity PA with static and awkward positions. Additionally, workers are predominantly women in both occupation groups. We need, however, to be careful when comparing the results from studies using objectively measured and self-reported PA [17]. Increasing evidence has proposed that OPA may not be health enhancing, but on the contrary, detrimental to health [8, 27]. This can be explained based on, for example, the long duration of OPA, the lack of sufficient recovery time, and lower worker control [27]. Our results with ECEC personnel match this evidence by suggesting that even light-intensity OPA may be considered overloading when it is long-lasting and occurring daily.

In this study, we did not observe any association between LTPA and the NFR. This finding contradicts the results of a previous study, which suggested that high LTPA, especially when of a high intensity, engenders a lower NFR [28]. Similarly, high-intensity PA during leisure time is reported to be associated with better WA [7, 21, 29, 43]. First, the conflicting results may be due to the different instruments used to measure PA as we used an accelerometer-based instrument in contrast to the instruments based on self-reporting used in many studies. Second, only very small amounts of moderate LTPA and almost a total lack of vigorous LTPA were detected in our participants. This may suggest that LTPA only works as a stress revealer with regard to MVPA. It can be speculated that if physical work demands are high compared to individuals’ capacity, there are no resources left over for LTPA [48].

Earlier studies have reported NFR scores (0–100) in office workers (mean = 27.3, SD = 29.6) [55] and cleaners (mean = 53.9, SD = 28.0) [39], for example. In our study, the mean NFR among ECEC professionals was 38.4 (SD = 26.2). It has been suggested that a score higher than 54.5 indicates a risk of psychological symptoms [55]. Coffeng et al. [11] studied office workers and reported that 77% of the population had a low NFR (< 54.5) and that 23% of the workers had a high NFR (> 54.5). In the industry and healthcare sectors, the results were 60%–80% and 20%–40%, respectively [20]. There is no earlier research about NFR level on ECEC professionals. Based on our data 67% of the participants had a low and 33% had a high NFR and the proportions are approximately at the same level as among healthcare workers [20].

Insufficient PA is a well-established health risk, and about 27% of the global population does not meet the WHO recommendations on PA for health [23]. Several national level papers report that only 10%–31% of the adult population meet both MVPA and strength-training recommendations [1–3, 25, 30]. When measured with an accelerometer, only about 25% of the adult Finnish population meets the criteria for health-enhancing PA. Individuals spend almost 11 h of their awake time lying, sitting, or standing still, 3 h on light PA, 42 min on moderate PA, and only a few minutes on vigorous PA [31]. Our results regarding total PA were in line with those of Husu et al.’s [30] findings: 9.2 h on sedentary behavior, 5.8 h on light PA, 46 min on moderate PA, and 2 min on vigorous PA. ECEC professionals’ lower sedentary times and higher light PA times when compared with the adult Finnish population might be explained by the amount of OPA. Occupational sedentary time in ECEC center work is scarce since the work comprises both inside play activities as well as outdoor activities with children. Among our participants, this also resulted in that the total PA times in terms of OPA and LTPA were quite similar, with the mean being about four hours/day for both. For adults, the recommendation for PA is 150 min per week of moderate intensity aerobic activity along with muscle strength training at moderate to high intensity twice a week [53]. There are no earlier results about the ECEC professionals’ PA levels but only about 30% of our participants fulfilled the strength-training recommendations. Lack of moderate and vigorous PA is also worth noticing. High level of OPA does not mean that PA recommendations are fulfilled [40]. It has been recognized that OPA is not always health-enhancing and adequate amount of LTPA would be required to achieve functional capacity that can help to cope with physical challenges at work.

One strength of this study was the objective measurement of PA. The majority of studies on the relationship between PA and the NFR have used self-reports to measure PA levels. These methods include the risk of bias because of their lack of accuracy and the tendency of participants to overestimate the amount of PA and underestimate the time spent on sedentary behavior [22, 45, 47]. Especially, the use of accelerometers has been infrequent in regard to OPA. However, accelerometers have some limitations as well. It is possible that the accelerometers were unable to capture some ECEC occupation-specific PA, including heavy tasks, like lifting children, or awkward and static positions when dressing or undressing small children, for example. Further, accelerometer measured LTPA includes not only specific exercise or workout sessions but for the most parts light intensity everyday life activities. Accelerometers can be a good tool for measuring the total PA during a certain time frame, but they might fail to detect different types of PA [17], for example strength training, yoga etc. Further, earlier studies on associations between the NFR and PA have either not separated OPA and LTPA or concentrated on OPA or LTPA. In this study, with the help of diaries, we were able to record working hours and bedtimes and separately analyze both domains of OPA and LTPA. Because of the cross-sectional study design, no causal relations could be stated. Also, we used a relatively large sample, but one needs to be careful when generalizing our results to men.

## Conclusions

To conclude, our results suggest that long-lasting light intensity OPA is associated with a high NFR and challenge recovery even without strenuous PA at work. In addition, low level and low intensity LTPA appear not to have relevance to the NFR. At ECEC centers, it might be useful to analyze the physical stress factors of specific work tasks and try to modify the work to better balance the different physical behaviors and their intensities to make them more health enhancing. Additionally, higher level and more intense PA during leisure time would be recommendable as it may increase physical capacity and stress resilience of ECEC workers.

## Data Availability

The data are available from the first author upon reasonable request and with permission of Finnish Institute of Occupational Health, Helsinki, Finland.
